# An elevated parametric thyroid feedback quantile-based index is associated with atrial fibrillation

**DOI:** 10.3389/fendo.2023.1087958

**Published:** 2023-02-23

**Authors:** Vanesa Alonso-Ventura, Patricia Campos-Magallon, Belen Moreno-Franco, Pilar Calmarza, Fernando Calvo-Gracia, Jose Manuel Lou-Bonafonte, Patricia de Diego-Garcia, Jose Antonio Casasnovas, Victoria Marco-Benedi, Fernando Civeira, Martin Laclaustra

**Affiliations:** ^1^Hospital Universitario Miguel Servet, Zaragoza, Spain; ^2^Instituto de Investigación Sanitaria de Aragón (IIS Aragón), Zaragoza, Spain; ^3^Facultad de Medicina, Universidad de Zaragoza, Zaragoza, Spain; ^4^Hospital San Pedro, Logroño, Spain; ^5^Centro de Investigación Biomédica en Red de Enfermedades Cardiovasculares-Instituto de Salud Carlos III (CIBERCV-ISCIII), Madrid, Spain; ^6^Hospital Clínico Universitario Lozano Blesa, Zaragoza, Spain; ^7^Instituto Agroalimentario de Aragón, CITA-Universidad de Zaragoza, Zaragoza, Spain; ^8^Centro de Investigación Biomédica en Red de Fisiopatología de la Obesidad y Nutrición-Instituto de Salud Carlos III (CIBEROBN-ISCIII), Madrid, Spain

**Keywords:** atrial fibrillation, thyroid regulation, thyrotropin, thyroxine, Parametric Thyroid Feedback Quantile-Based Index, PTFQI

## Abstract

**Introduction:**

Atrial fibrillation is associated with hyperthyroidism. Within the euthyroid range, it is also associated with high thyroxine (fT4), but not with thyrotropin (TSH). We aim to describe differences in thyroid regulation, measured by the Parametric Thyroid Feedback Quantile-Based Index (PTFQI), between patients with atrial fibrillation and the general population.

**Materials and methods:**

Thyroid parameters (PTFQI, TSH, and fT4) of a sample of 84 euthyroid subjects with atrial fibrillation (cases) were compared to a reference sample of euthyroid healthcare patients (controls). We calculated age and sex adjusted ORs for atrial fibrillation across tertiles of these parameters. Also, within cases, we studied thyroid parameters association with clinical characteristics of the atrial fibrillation.

**Results:**

After adjusting for age and sex, fT4 and PTFQI were higher in subjects with atrial fibrillation when compared to the general sample (p<0.01 and p=0.01, respectively). Atrial fibrillation ORs of the third versus the first PTFQI tertile was 1.88(95%CI 1.07,3.42), and there was a gradient across tertiles (p trend=0.02). Among atrial fibrillation patients, we observed that higher PTFQI was associated with sleep apnea/hypopnea syndrome (OSAS) (p=0.03), higher fT4 was associated with the presence of an arrhythmogenic trigger (p=0.02) and with heart failure (p<0.01), and higher TSH was also associated with OSAS (p<0.01).

**Conclusions:**

Euthyroid subjects with atrial fibrillation have an elevation of the pituitary TSH-inhibition threshold, measured by PTFQI, with respect to the general population. Within atrial fibrillation patients, high PTFQI was associated with OSAS, and high fT4 with heart failure. These results hint of the existence of a relationship between thyroid regulation and atrial fibrillation.

## Introduction

Atrial fibrillation is the most common cardiac arrhythmia in our setting (1, 2). Thyroid disorders and, in particular, hyperthyroidism are a recognized risk factor for atrial arrhythmias (2–5), which have to be considered among other conditions that pose a risk for developing atrial fibrillation (smoking, diabetes mellitus, hypertension, coronary heart disease, heart failure, left atrial enlargement, or obesity) (6).

Similarly to patients with thyroid disease, in euthyroid subjects (normal values of thyrotropin -TSH- and free thyroxine -fT4-), fT4 levels in the higher normal range are associated with the development of atrial fibrillation (7–9). However, TSH levels are not so clearly associated with the development of atrial fibrillation: on the one hand, there are studies that conclude that there is no association (7, 8, 10) and, on the other hand, there are studies that suggest a higher risk of developing atrial fibrillation with TSH values close to the lower normal limit (9, 11, 12). High fT4 is most often present in the context of primary thyroid disease (13), where TSH tends to be suppressed. The controversy in previous studies suggests that primary thyroid disease justify only part of the cases in which high fT4 is associated with atrial fibrillation. Thus, investigating whether altered thyroid regulation is related to atrial fibrillation, beyond the already known effects of primary hyperthyroidism, becomes relevant. Indeed, our previous results (14) encourage researching this association. Thyroid regulation can be studied with biochemical indexes. In particular, the Parametric Thyroid Feedback Quantile-Based Index (PTFQI) (14, 15) quantifies the deviations from the physiological population-average pituitary response to thyroid hormone and ranges from -1 to 1: negative values indicate an abnormally low TSH for fT4 values (a down-regulated TSH-inhibition threshold) and positive values indicate an abnormally high TSH given fT4 levels (an up-regulated TSH-inhibition threshold).

The main objective of this work is to describe differences in thyroid regulation and thyroid hormone levels between euthyroid subjects with atrial fibrillation and the general population, as well as to investigate the association of these parameters with clinical conditions that favor it or worsen its prognosis.

## Materials and methods

### Design and subjects

Thyroid parameters (PTFQI, TSH, and fT4) of a sample of patients with atrial fibrillation were compared to a reference sample of healthcare patients (*case-control study*). Patients with atrial fibrillation (paroxistic or persistent) who required, at least, one hospital admission were included as cases. The control sample PTFQI distribution tertiles were used as cut-offs for dividing cases and controls. We compared atrial fibrillation odds across the tertiles.

Within the cases sample, additionally, a cross-sectional study of association of the thyroid parameters with clinical characteristics was conducted (*cross-sectional study*).

We reviewed the 165 clinical records of the patients with atrial fibrillation who were admitted to the Miguel Servet University Hospital in Zaragoza (Spain) from July 2017 to June 2019. Exclusion criteria included previous thyroid disease or abnormal TSH and fT4 (which was the main cause of exclusion, 72%) and diseases or pharmacological treatments that interfere with the thyroid axis, among which amiodarone use accounted for the 35% of excluded pacients (**Supplementary Material Table 1**). Only patients who had TSH and fT4 values available before medical intervention (electrical cardioversion, catheter ablation, or treatment with amiodarone) were included. The number of patients identified for the analysis was 84 (**Supplementary Material Figure 1**).

Thyroid parameters of healthcare patients older than 18 years who underwent a thyroid hormone measurement across three months of 2018, in the central laboratory of the Miguel Servet University Hospital (6051 patients) were used to calculate the reference parameters for the PTFQI formula (14). Euthyroid participants of this sample constituted the control group (n=5256).

This study protocol was approved by the Ethical Committee of Clinical Research of Aragón (CEICA) (expedient numbers 19-041 and 19-519) who authorized review of clinical records.

### Laboratory measurements and methods

TSH and fT4 were measured in an automated analyzer by Unicel DXi Beckman’s System^®^. Their normal ranges were 0.38-5.33 µUI/mL for TSH and 7.46-21.11 pmol/L for fT4.

### Parametric thyroid feedback quantile-based index

The PTFQI (14, 15) is calculated with a formula: ϕ ((fT4 - µ_fT4_)/σ_fT4_) - (1-ϕ ((ln TSH - µ_ln TSH_)/σ_ln TSH_))), where µ_fT4_ = 11.49 pmol/L, σ_fT4_ = 2.46 pmol/L, µ_ln TSH_ = 0.55, and σ_ln TSH_ = 1.00 in our reference sample (see above).

### Clinical data of atrial fibrillation patients

Demographics (sex, age), clinical diagnoses (atrial fibrillation triggers, heart failure, obstructive sleep apnea/hypopnea syndrome -OSAS-) and echocardiographic findings (atrial enlargement, heart valve disease) were retrieved from clinical records. Obesity was defined as BMI ≥ 30 kg/m^2^.

### Statistical analysis

Continuous variables were described as mean and SD, and categorical variables as percentage and count. Linear and logistic regression models were fitted to estimate differences and ORs.

The control sample PTFQI distribution tertiles were used to divide cases and controls into three groups. We compared case and control groups, after adjusting for age and sex.

Within the cases sample, the association of the three thyroid parameters (PTFQI, TSH, and fT4) with dichotomous characteristics of their atrial fibrillation was also studied. These models were additionally adjusted for type 2 diabetes and obesity.

All analyses were performed with statistical computing software R version 4.1.

## Results

Among the 165 clinical records of atrial fibrillation reviewed, after applying the exclusion criteria, 51% were included for further analysis (**Supplementary Material Table 1**). Clinical records of those 84 euthyroid subjects (47.6% men) with a mean age of 70.3 (14.3) years were analyzed. The mean age of their first atrial fibrillation episode was 69.1 (14.6) years (**Supplementary Material Tables 2**, **4**).

### Thyroid parameters comparison between subjects with atrial fibrillation and the general sample

The mean age of cases was higher than that of controls (70.3 vs. 58.5 years, p<0.01) and the proportion of men in the general sample was lower (47.6% vs. 36.1%, p=0.03). In the adjusted model, we found that fT4 and PTFQI were higher in subjects with atrial fibrillation when compared to the general sample (p<0.01 and p=0.01, respectively) (**Table 1**). However, despite not significantly, TSH was higher in the atrial fibrillation sample, contrary to the expected lower TSH in primary thyroid disorders with fT4 elevation.

**Table 1:** Thyroid parameters conditionally-adjusted means for mean age 70 years and a proportion of men 50%.

Variable	General Sample	Atrial Fibrillation Sample	Difference (95% CI)	p
PTFQI	0.04	0.12	0.08 (0.02,0.14)	0.01 *
TSH geometric mean	0.62	0.65	0.03 (-0.09,0.15)	0.61
fT4	11.74	12.33	0.59 (0.17,1.01)	< 0.01 *

Mean difference (95% CI) from a linear regression model adjusted for age and sex. * denotes p < 0.05.

PTFQI, Parametric Thyroid Feedback Quantile-based Index.

**Figure 1:**
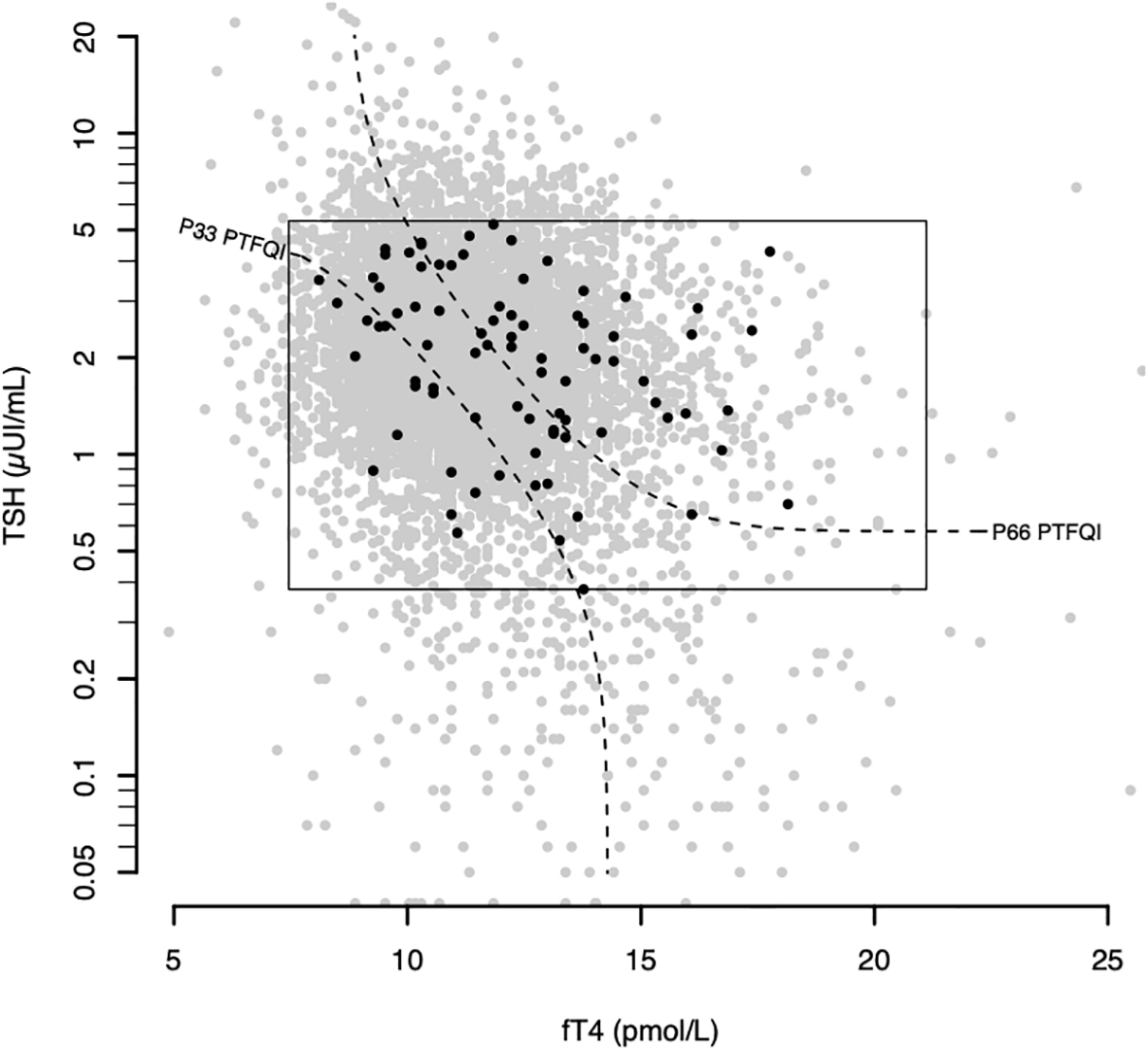
Atrial fibrillation plotted over the general sample in the thyroid regulation space. Gray dots represent all thyroid analyses between September and November of 2018 (6434 samples). The rectangle defines the normal values. Black dots represent atrial fibrillation subjects (cases). Black dashed curves represent the 33rd and 66th percentiles of the control group PTFQI dividing it in 3 equal parts. fT4 – thyroxine; TSH – thyrotropin; PTFQI-Parametric Thyroid Feedback Quantile-based Index; P number- Percentile number.

Atrial fibrillation cases count grew across higher PTFQI tertiles (**Figure 1**). Adjusted ORs of the third versus the first PTFQI tertile for atrial fibrillation was 1.88 (95% CI 1.07,3.42) (p trend=0.02) (**Table 2**). Conversely, there was not a clear atrial fibrillation adjusted OR gradient across TSH tertiles nor fT4 tertiles (**Supplementary Material Table 5**).

**Table 2:** OR (95% CI) referenced to the first tertile PTFQI-group, adjusted for age and sex.

Atrial Fibrillation	PTFQI tertiles			p trend
	1^st^	2^nd^	3^rd^	
	[min., -0.136]	(-0.136, 0.131]	(0.131, max.]	
**n_AF_ vs n_C_**	17 vs 1754	24 vs 1750	43 vs 1752	0.02 *
**OR**	1.00	1.25	1.88	
	(reference)	(0.67, 2.38)	(1.07,3.42)	

PTFQI, Parametric Thyroid Feedback Quantile-based Index; n_AF_, number of atrial fibrillation cases; n_C_, number of controls. * denotes p < 0.05. P trend calculated entering PTFQI tertile as a continuous variable.

### Thyroid parameters among atrial fibrillation patients

After adjusting for age, sex, diabetes, and obesity, we observed a relationship between thyroid parameters (PTFQI, TSH, and fT4) and some clinical features. Among atrial fibrillation subjects, those who presented any of the clinical characteristics studied (atrial enlargement, heart valve disease, arrhythmogenic trigger, heart failure, OSAS) had a numerically higher PTFQI, although statistical significance was only reached in OSAS group (p=0.03) (**Table 3**).

**Table 3:** PTFQI, TSH and FT4 means among. subjects with paroxistic or persistent atrial fibrillation by clinical characteristics.

Variable		Absent	Present	p
Atrial enlargement	n/N	33/84	51/84	
PTFQI	Mean(SD)	0.09 (0.29)	0.15 (0.28)	0.44
TSH	Geometric mean	1.76	2.10	0.11
fT4	Mean(SD)	0.97 (0.17)	0.95 (0.19)	0.33
Heart valve disease	n/N	43/84	41/84	
PTFQI	Mean(SD)	0.09 (0.27)	0.16 (0.30)	0.45
TSH	Geometric mean	1.91	2.00	0.37
fT4	Mean(SD)	0.94 (0.14)	0.98 (0.21)	0.99
Arrhythmogenic trigger	n/N	80/84	4/84	
PTFQI	Mean(SD)	0.11 (0.28)	0.35 (0.21)	0.12
TSH	Geometric mean	1.97	1.68	0.66
fT4	Mean(SD)	0.95 (0.17)	1.16 (0.17)	0.02 *
Heart failure	n/N	67/84	17/84	
PTFQI	Mean(SD)	0.10 (0.28)	0.24 (0.27)	0.10
TSH	Geometric mean	2.03	1.70	0.77
fT4	Mean(SD)	0.93 (0.16)	1.09 (0.20)	< 0.01 *
Obstructive sleep apnea/hypopnea syndrome	n/N	79/84	5/84	
PTFQI	Mean(SD)	0.11 (0.28)	0.39 (0.32)	0.03 *
TSH	Geometric mean	1.81	4.04	< 0.01 *
fT4	Mean(SD)	0.96 (0.17)	0.99 (0.26)	0.75

Data expressed as mean (SD). p value from a regression linear model. Data adjusted for age, sex, diabetes, and obesity. * denotes p < 0.05.

PTFQI, Parametric Thyroid Feedback Quantile-based Index.

Significant differences in fT4 were found in the presence of an arrhythmogenic trigger (p=0.02) and heart failure (p<0.01). Patients with these characteristics had slightly lower, although not statistically significant, TSH values. In the remainder characteristics considered, TSH was numerically higher, reaching statistical significance for subjects with OSAS (p<0.01) (**Table 3**).

## Discussion

The major finding in this study was that patients with atrial fibrillation had a significantly higher PTFQI when compared to the general population. While high-normal fT4 levels had been previously associated with atrial fibrillation development (11), this is the first time that thyroid central regulation measured by PTFQI in atrial fibrillation patients is compared to that in the general population. Recognizing the importance of thyroid regulation on atrial fibrillation may provide tools for atrial fibrillation prevention strategies.

Among atrial fibrillation subjects, those with OSAS had higher PTFQI and TSH, while those with an arrhythmogenic trigger and heart failure had higher fT4 levels.

### TSH and fT4 and atrial fibrillation odds

The association between TSH within its normal range and atrial fibrillation is controversial in both new-onset and recurrent atrial fibrillation (11, 16). In our study, we found similar TSH concentrations in cases and controls, although they were slightly higher, despite non-significant, in atrial fibrillation cases. If most triggering effects would depend on raised fT4 due to primary thyroid disease, a lower TSH would be expected among atrial fibrillation patients.

The evidence regarding fT4 and its relationship with atrial fibrillation is more consistent, and it is present even without hyperthyroidism. In fact, higher fT4 levels within the reference range were associated with an increased prevalence and incidence of atrial fibrillation, which could represent a challenge to the diagnostic paradigm of this entity (7). The mechanism of fT4 impact on atrial fibrillation risk might be explained by the increase in vascular resistance, cardiac contractility, heart rate, and atrial automaticity that thyroid signaling stimulates (7).

Thus, our separate analyses of TSH and fT4 suggest that levels of both variables are higher in subjects with atrial fibrillation when compared to the general population, although the most important effect is that of fT4. The absence of TSH inhibition leads to delve deeper in the analysis of regulation.

### Thyroid regulation and atrial fibrillation odds

Here, we described for the first time that those patients with atrial fibrillation had higher PTFQI (meaning a concomitant elevation of TSH and fT4, which indicates a higher pituitary TSH-inhibition threshold) despite being euthyroid. Assuming that fT4 and TSH were at equilibrium for all participants (17), the observed deviation from the average equilibrium in the population may mean, either a normal or high-normal thyroid secretory capacity together with a decreased hypothalamus-pituitary sensitivity to fT4, or an elevation of TSH secretion accompanied by a decreased thyroid secretory capacity (18), both implying that TSH inhibition is decreased with respect to the population average. There is a known association of atrial fibrillation with high normal fT4, which must stem from a high functioning thyroid, a higher secretion of TSH, or both. Given that we found higher PTFQI, the latter mechanism seems more probably responsible. Therefore, central up-regulation of the thyroid axis might be relevant in this atrial arrhythmia.

Attending to the different distribution of thyroid hormone receptors (19) (beta-receptor predominant in thyrotrophic cells and alpha-receptor predominant in myocytes), a reduced pituitary sensitivity to fT4 might be involved consistently in the development of atrial fibrillation: while fT4 levels are not enough to inhibit TSH release at the pituitary level, they are enough to exert possible deleterious effects on the heart.

Although this hypothesis could explain the relation of higher PTFQI with atrial fibrillation development, a cause-effect relationship cannot be established with the present study and the mechanism that relate this pituitary TSH-inhibition threshold elevation and atrial fibrillation should be elucidated in future research.

### Thyroid parameters and atrial fibrillation clinical characteristics

We found higher fT4 among atrial fibrillation patients with heart failure, an association that to our knowledge had not been previously described. A general association of high fT4 and heart failure was already known (20), and one of the mechanisms that explains it is that fT4 favors atrial fibrillation. Given that all the patients had atrial fibrillation, the association described must depend on other factors beyond fT4 arrhythmogenicity. Higher thyroxine initially stimulates contractility and cardiac output but longstanding thyrotoxicosis leads to a decrease of these parameters and to heart failure (21, 22). Although our study includes only euthyroid patients, similar mechanisms could be at stake.

Within atrial fibrillation patients, PTFQI does not seem to be particularly associated with atrial enlargement, heart valve disease, arrhythmogenic trigger, or heart failure, but we found association with OSAS, where the pituitary TSH-inhibition threshold was substantially higher. Previous studies have shown that thyroid dysfunction is associated with sleep disorders (23). Hypothyroidism may play a role in the development of OSAS, although the specific pathophysiologic mechanism remains elusive (23). Despite high PTFQI does not resemble a hypothyroid condition, insufficient action of thyroid hormone (peripheral resistance) might occur in some peripheral tissues, mimicking hypothyroid-like effects. Also, OSAS, or conditions that appear in the OSAS context, like obesity, may induce changes in the thyroid regulation. However, the small number of OSAS patients in our study dictates prudence in interpreting this result. It will be of interest to investigate in the future if this is one mechanism that connects both diseases.

### Future prevention strategies

Adequate control of hyperthyroidism helps preventing atrial fibrillation (2). In addition, thyroid treatment in atrial fibrillation patients with overt hyperthyroidism has been proposed in several studies not only for better control but also for avoiding recurrences (2, 24). In the specific case of euthyroid subjects, given the association of high levels of fT4 with the development of atrial fibrillation, the need for the redefinition of normal limits has been proposed (7). However, beyond this, according to our results, the thyroid regulation measured by PTFQI could help to detect subjects at risk of developing atrial fibrillation. Also, current cost-saving strategies that promote measuring only TSH to evaluate thyroid function may need to be reconsidered, at least in the arrhythmology field. Furthermore, adjusting treatments to normalize PTFQI might be a new opportunity to be addressed in future research.

### Limitations

This study has several limitations. First, since the study included a general sample of healthcare patients who, for different reasons underwent a biochemical analysis, all results must be carefully interpreted because this sample is not fully representative of the general population, but of healthcare patients. In addition, patients with atrial fibrillation were not removed from the general sample (as that information was not available) which leads to a bias towards the null of our results due to misclassification error. Nonetheless, this has a limited impact, as we assume that only a very small fraction of the reference population had atrial fibrillation. Also, considering the direction of the bias, we still were able to demonstrate several associations with statistical significance, so true differences can be assumed to be larger than those described here. Second, the modest sample size implies that statistical significance may not have been reached in some analyses in spite of an apparent tendency in the associations. Third, fT3 levels were not available, so its effects on thyroid regulation and cardiomyocytes could not be studied. Finally, only thyroid parameters and not detailed clinical characteristics were available for the general sample limiting study of other different conditions between both groups.

## Conclusions

In conclusion, euthyroid subjects with atrial fibrillation have an elevation of the pituitary TSH-inhibition threshold, measured by PTFQI, with respect to the general population. Although higher fT4 levels were observed among them, a TSH inhibition consistent with primary thyroid disorders was not present. Within atrial fibrillation patients, high PTFQI was associated with OSAS, and high fT4 with heart failure. These results hint of the existence of a relationship between the thyroid regulation and atrial fibrillation.

## Data availability statement

The original contributions presented in the study are included in the article/**Supplementary Material**. Further inquiries can be directed to the corresponding author.

## Ethics statement

This study protocol was approved by the Ethical Committee of Clinical Research of Aragón: CEICA, Instituto Aragonés de Ciencias de la Salud, Avda, San Juan Bosco, 13. 50009, Zaragoza, España (expedient numbers 19-041 and 19-519) who authorized review of clinical records.

## Author contributions

VA-V and ML conceptualized the research question and designed the study. VA-V and ML performed the statistical data analysis. VA-V, BM-F, JML-B and ML, interpreted the results and VA-V and ML wrote the manuscript. PC-M, PC, FC-G, JML-B, PD-G, JAC, VM-B, and FC revised the manuscript for important intellectual content. All authors contributed to the article and approved the submitted version.
