# Supplementary material for: An elevated parametric thyroid feedback quantile-based index is associated with atrial fibrillation
Source: Front Endocrinol (Lausanne). 2023 Feb 23;14:1087958. doi: 10.3389/fendo.2023.1087958 (PMC9995977; doi:10.3389/fendo.2023.1087958)
Supplement: Supplementary file 1 [file Presentation_1.pdf]

Extended results of a case-control study. An elevated Parametric Thyroid Feedback Quantile-Based Index is associated with atrial fibrillation

Alonso-Ventura V, Campos-Magallon P, Moreno-Franco B, Calmarza P, Calvo-Gracia F, Lou-Bonafonte JM, de Diego-Garcia P, Casasnovas JA, Marco-Benedi V, Civeira F, Laclaustra M.

Frontiers 2023

This report supplements the results published at:

Alonso-Ventura V, Campos-Magallon P, Moreno-Franco B, Calmarza P, Calvo-Gracia F, Lou-Bonafonte JM, de Diego-Garcia P, Casasnovas JA, Marco-Benedi V, Civeira F and Laclaustra M (2023) An elevated parametric thyroid feedback quantile-based index is associated with atrial fibrillation. Front. Endocrinol. 14:1087958.doi: 10.3389/fendo.2023.1087958

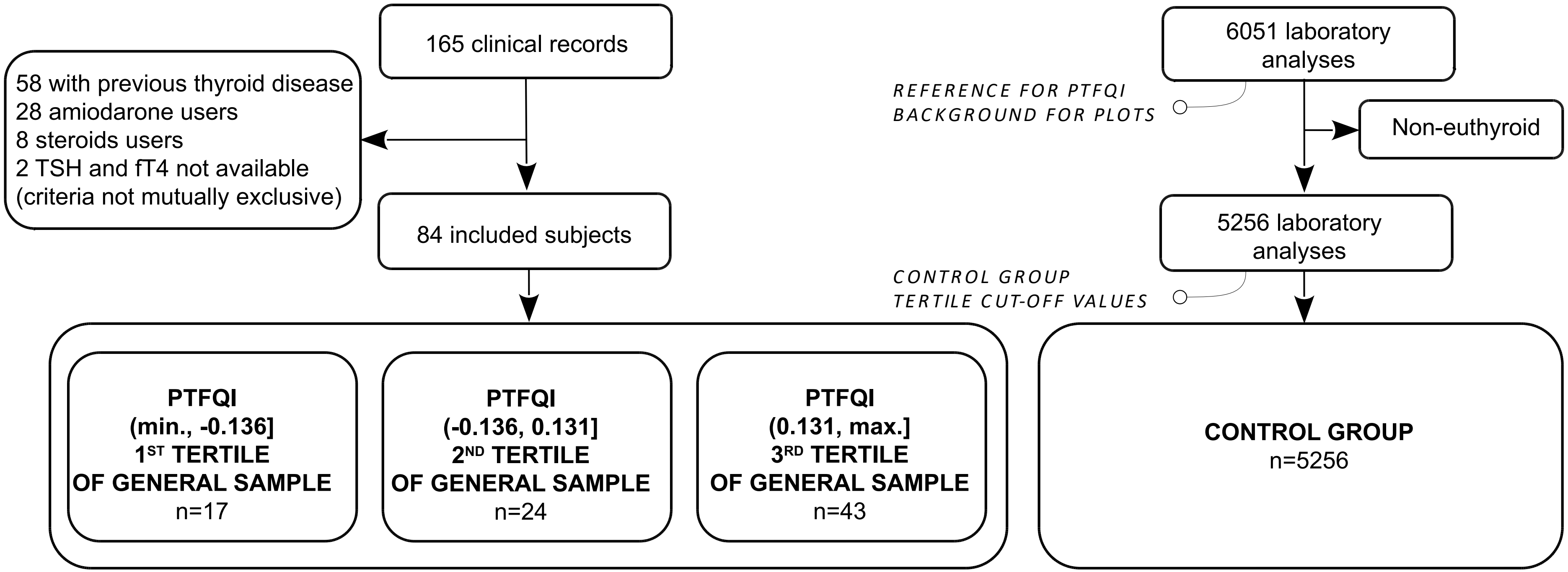

Figure 1. Flow chart.

We reviewed the 165 clinical records of the patients with atrial fibrillation who were admitted to the Miguel Servet University Hospital in Zaragoza (Spain) from July 2017 to June 2019. Exclusion criteria are shown in the next page. Only patients who had TSH and fT4 values available before medical intervention were included. These patients were compared with healthcare patients older than 18 years who underwent a thyroid hormone measurement across three months of 2018, in the same hospital. Only euthyroid participants of this sample constituted the control group.

EXCLUSION CRITERIA	N excluded
	n/N
Total excluded	81/165
Previous thyroid disease	58/165
Previous biochemical alteration	35/165
Clinical hyper- or hypothyroidism	30/165
Treatment with levothiroxine	26/165
Radioactive iodine treatment	2/165
Treatment with amiodarone (at least one year before)	28/165
Treatment with steroids	8/165
TSH and fT4 values not available	2/165

Table 1. Exclusion criteria ordered by relevance. Criteria are not mutually exclusive.

			PTFQI tertiles			
			1st	2nd	3rd	p heter
		Overall	[min., -0.136]	(-0.136, 0.131]	(0.131, max.]	
	n	84	17	24	43	
**Age**	84	70.3(14.3)	70.6(13.5)	68.9(14.6)	71.0(14.8)	0.850
**Sex (women)**	84	52.4[44]	58.8[10]	50.0[12]	51.2[22]	0.827
**Weight**	71	75.7(15.8)	75.4(18.1)	77.4(12.8)	74.8(16.8)	0.887
**Age of first episode**	82	69.1(14.6)	68.9(16.9)	68.9(11.7)	69.3(15.3)	0.649
**Obesity**	84	31.0[26]	23.5[4]	41.7[10]	27.9[12]	0.408
**Diabetes**	84	22.6[19]	17.6[3]	29.2[7]	20.9[9]	0.555
**Atrial enlargement**	84	60.7[51]	64.7[11]	50.0[12]	65.1[28]	0.498
**Heart valve disease**	84	48.8[41]	47.1[8]	54.2[13]	46.5[20]	0.663
**Arrhythmogenic trigger**	84	4.8[4]	0.0[0]	0.0[0]	9.3[4]	0.065
**Heart failure**	84	20.2[17]	11.8[2]	13.6[3]	27.9[12]	0.252
**Obstructive sleep apnea/hypopnea síndrome**	84	6.0[5]	0.0[0]	4.5[1]	9.8[4]	0.256

Table 2. Description of the sample of subjects with atrial fibrillation across PTFQI tertiles. Data expressed in mean (standard deviation) and in percentage [number]. P-heter is the p value for testing differences among groups, unadjusted. PTFQI: Parametric Thyroid Feedback Quantile-based index.

			TSH tertiles			
			1st	2nd	3rd	p heter
		Overall	[min.,0.412]	(0.412,0.904]	(0.904,max.]	
	n	84	29	21	34	
**Age**	84	70.3(14.3)	73.4(14.0)	71.6(13.5)	66.9(14.8	0.141
**Sex (women)**	84	52.4[44]	58.6[17]	42.9[9]	52.9[18]	0.431
**Weight**	71	75.7(15.8)	74.2(13.8)	77.3(18.6)	75.7(15.7)	0.977
**Age of first episode**	82	69.1(14.6)	71.9(15.1)	70.4(13.9)	65.7(14.4)	0.259
**Obesity**	84	31.0[26]	23.5[4]	41.7[10]	27.9[12]	0.408
**Diabetes**	84	22.6[19]	17.6[3]	29.2[7]	20.9[9]	0.555
**Atrial enlargement**	84	60.7[51]	41.4[12]	81.0[17]	64.7[22]	0.006
**Heart valve disease**	84	48.8[41]	37.9[11]	66.7[14]	47.1[16]	0.055
**Arrhythmogenic trigger**	84	4.8[4]	6.9[2]	0.0[0]	5.9[2]	0.311
**Heart failure**	84	20.2[17]	24.1[7]	20.0[4]	18.2[6]	0.976
**Obstructive sleep apnea/hypopnea síndrome**	84	6.0[5]	0.0[0]	0.0[0]	15.2[5]	0.005

Table 3. Description of the sample of subjects with atrial fibrillation across TSH tertiles. Data expressed in mean (standard deviation) and in percentage [number]. P-heter is the p value for testing differences among groups, unadjusted.

			fT4 tertiles			
			1st	2nd	3rd	p heter
		Overall	[min.,10.4]	(10.4,12.0]	(12.0,max.]	
	n	84	21	19	44	
**Age**	84	70.3(14.3)	67.7(14.7)	68.3(13.8)	72.4(14.4)	0.163
**Sex (women)**	84	52.4[44]	57.1[12]	57.9[11]	47.7[21]	0.297
**Weight**	71	75.7(15.8)	77.2(16.4)	78.1(15.7)	73.9(15.8)	0.511
**Age of first episode**	82	69.1(14.6)	66.5(14.6)	67.8(14.5)	70.8(14.8)	0.007
**Obesity**	84	31.0[26]	23.5[4]	41.7[10]	27.9[12]	0.408
**Diabetes**	84	22.6[19]	23.8[5]	21.1[4]	22.7[10]	0.885
**Atrial enlargement**	84	60.7[51]	66.7[14]	57.9[11]	59.1[26]	0.628
**Heart valve disease**	84	48.8[41]	57.1[12]	36.8[7]	50.0[22]	0.328
**Arrhythmogenic trigger**	84	4.8[4]	0.0[0]	5.3[1]	6.8[3]	0.301
**Heart failure**	84	20.2[17]	10.5[2]	15.8[3]	27.3[12]	0.432
**Obstructive sleep apnea/hypopnea síndrome**	84	6.0[5]	5.3[1]	10.5[2]	4.8[2]	0.497

Table 4. Description of the sample of subjects with atrial fibrillation across fT4 tertiles. Data expressed in mean (standard deviation) and in percentage [number]. P-heter is the p value for testing differences among groups, unadjusted. Sex is expressed as women percentage.

Atrial Fibrillation	TSH tertiles			p trend
	1st	2nd	3rd	
	[min.,0.412]	(0.412,0.904]	(0.904, max.]	
n_{AF} vs n_C	29 vs 1765	21 vs 1743	34 vs 1748	
OR	1.00	0.80	1.23	0.42
	(reference)	(0.45, 1.41)	(0.74, 2.04)	

Atrial Fibrillation	fT4 tertiles			p trend
	1st	2nd	3rd	
	[min.,10.4]	(10.4,12.0]	(12.0, max.]	
n_{AF} vs n_C	21 vs 1832	19 vs 1704	44 vs 1720	
OR	1.00	0.84	1.56	0.06
	(reference)	(0.44 1.58)	(0.92, 2.72)	

Table 5. OR (95% CI) referenced to the first tertile group, adjusted for age and sex. P trend calculated entering tertile as a continuous variable.

n_{AF}: number of atrial fibrillation cases. $n_{C:}$ number of controls.

While adjusted ORs for atrial fibrillation were 1.25 (95% CI 0.67,2.38) for the second PTFQI tertile and 1.88 (95% CI 1.07,3.42) for the third PTFQI tertile versus the first one, with a statistically significant linear trend (p=0.02), there was not a clear OR gradient across TSH tertiles nor fT4 tertiles.
